# A patient with myotonic dystrophy diagnosed after experiencing sudden respiratory failure: a case report

**DOI:** 10.1186/s40981-020-00388-7

**Published:** 2020-10-08

**Authors:** Noriaki Nishihara, Shunsuke Tachibana, Hajime Sonoda, Michiaki Yamakage

**Affiliations:** 1 Department of Anesthesiology, Sapporo Medical University School of Medicine, South 1, West 16, Chuo-ku, Sapporo, Hokkaido 060-8543 Japan; 2 Department of Anesthesiology, Kushiro City General Hospital, Shunkodai 1-12, Kushiro, Hokkaido 085-0822 Japan

**Keywords:** Myotonic dystrophy, Respiratory failure, Ventricular fibrillation, Genetic test

## Abstract

**Background:**

Myotonic dystrophy is a disorder affecting multiple organs including skeletal muscles and causes respiratory failure. We describe a patient who developed respiratory failure, with delayed diagnosis of myotonic dystrophy type 1 as the cause.

**Case presentation:**

A 62-year-old woman developed acute onset of dyspnea after showing hypertension and tachycardia and was transported to our hospital. On arrival at our institution, SpO_2_ was 80% with a non-rebreather mask. With a diagnosis of acute phase heart failure, she underwent tracheal intubation. However, weaning from the respirator was difficult in the intensive care unit (ICU). A detailed interview revealed that her brother was affected with myotonic dystrophy type 1. She was also diagnosed with myotonic dystrophy type 1 by a genetic test.

**Conclusions:**

Taking a careful past and family history and prompt genetic testing is required on suspicion of neuromuscular diseases in a patient with respiratory failure by an unknown cause.

## Background

Myotonic dystrophy is the most common and the most severe form of myotonic syndrome, and it is divided into two main types: myotonic dystrophy type 1 and myotonic dystrophy type 2. Myotonic dystrophy type 1 is caused by a trinucleotide cytosine-thymine-guanine (CTG) repeat expansion in the dystrophia myotonica protein kinase (DMPK) gene. Myotonic dystrophy type 1 affects some muscles, such as facial muscles, levator palpebrae superioris, temporalis, sternocleidomastoids, distal muscles of the forearm, and hand intrinsic muscles. Moreover, myotonic dystrophy type 1 can also affect the eyes, heart, central nervous system (CNS), and endocrine system [1]. In general, the symptoms appear at a younger age, and molecular genetic testing for DMPK is necessary for a definite diagnosis of myotonic dystrophy. There are few reports of newly diagnosed with myotonic dystrophy type 1 in middle age. In this case report, a case of myotonic dystrophy type 1 that was newly diagnosed in middle age following sudden severe respiratory failure with difficulty in extubation is presented.

## Case presentation

Written, informed consent was obtained from the patient for genetic testing and for this case report. A 62-year-old woman (height: 152 cm, weight: 68.5 kg, BMI: 29.6 kg/m^2^) developed acute onset of dullness and dyspnea after showing hypertension (blood pressure 210/110 mmHg) and tachycardia (heart rate 124 beats per minute) and was transported to our hospital. Her history included non-treated asthma and uncontrolled diabetes mellitus (HbA1c: 7.5%). On arrival at our institution, she had critical respiratory failure (respiratory rate 30/min, unclear lung sounds, and SpO_2_ of 80% with a non-rebreather mask on 10 L/min of oxygen), requiring tracheal intubation. Followed by an increase of SpO_2_ to 100% and she was admitted to the ICU. Despite a lack of findings suggesting congestive heart failure on the chest X-ray, cardiologists initially suspected acute phase heart failure because of the preceding tachycardia and hypertension. On the second ICU day, ventricular fibrillation (Vf) occurred following QT prolongation, which required defibrillation. Emergency coronary angiography revealed only takotsubo syndrome. She was successfully weaned from artificial ventilation and the tracheal tube was removed on the 5th ICU day. However, she abruptly complained of dyspnea accompanied with a decrease of SpO_2_ to 90% with a non-rebreather mask on 10 L/min of oxygen, and she required reintubation on the 8th day. Laboratory data were unremarkable and there were no abnormalities indicative of heart failure or pulmonary diseases on chest X-ray except for mild atelectasis.

**Fig. 1:**
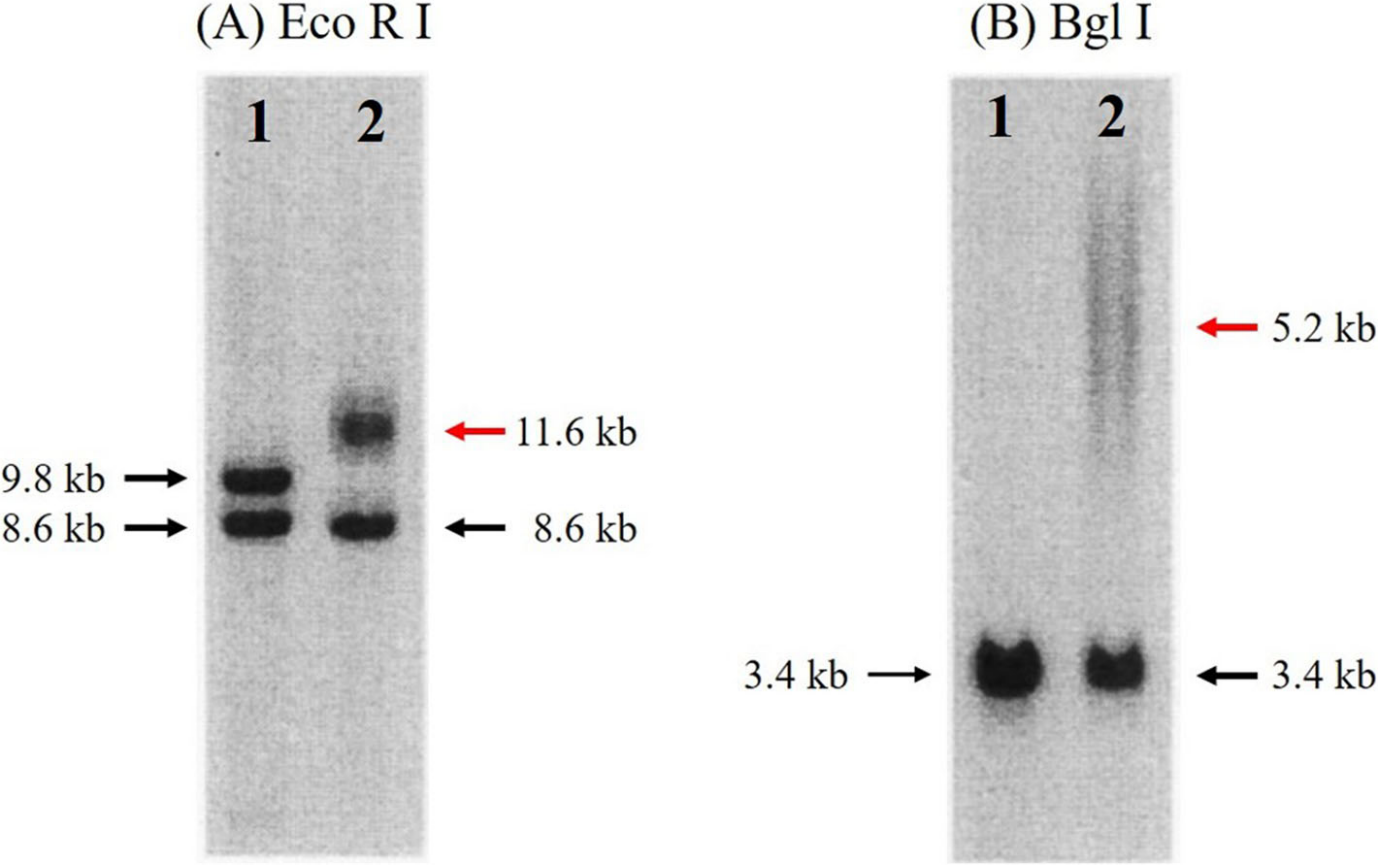
Southern blot analysis of the patient’s genomic DNA samples. Southern blots of (**a**) EcoRI- and (**b**) BglI-digested genomic DNA from a normal subject (control, lane 1) and from the myotonic dystrophy type 1 patient (case, lane 2). Expanded CTG repeat (red arrow) at 11.6 kb in DMPK gene was respectively detected in the affected patient

Because the cause of respiratory failure was not clear, the cardiologists consulted with our department. After re-taking her family history and past history carefully, several important episodes that contributed to the diagnosis were uncovered: (1) the patient required artificial ventilation for several days after general anesthesia in the past; (2) she had been complaining of weakness of bilateral upper limb muscles for a long time; (3) dysphagia had started a few months ago; and (4) her brother had been diagnosed with myotonic dystrophy type 1. Thus, her respiratory failure and muscle weakness were suspected to be the results of myotonic dystrophy. On the 26th day, a genetic test was performed on the patient’s blood samples (Fig. 1). The estimated number of CTG repeats in the DMPK gene was abnormally expanded to about 600 repeats, suggesting a diagnosis of the classical type of myotonic dystrophy type 1. After tracheotomy on the 34th day, she recovered from respiratory failure and was discharged from the ICU. She was transferred to a special hospital with no respiratory support.

## Discussion

In this report, a case of sudden and uncontrolled respiratory failure with undiagnosed myotonic dystrophy type 1 was presented. On suspicion of myotonic dystrophy based on her past and family history, the final diagnosis of myotonic dystrophy type 1 was made based on the results of the genetic test. Myotonic dystrophy type 1 is clinically categorized into three phenotypes (Table 1) [2]. Among the three phenotypes of myotonic dystrophy type 1, this case was classified as the classical type from the estimated number of CTG repeats (Table 1). Several reviewers have shown that myotonic dystrophy type 1 develops at a relatively young age and has a poor prognosis [1–3]. Especially in the classical type, the onset is generally at 10 to 30 years of age, and the average age at death is 48 to 60 years (Table 1). The present case therefore appeared to be rare, because the main symptom occurred at the age of 62 years, after middle age [3].

**Table 1:** Summary of the clinical phenotypes and CTG repeat lengths in myotonic dystrophy type 1

Phenotype	Clinical signs	CTG repeat size	Age of onset	Average age of death
			(years)	(years)
Mild	Cataracts	50 ~ 150	20 ~ 70	60 ~
	Mild myotonia			
Classical	Muscle weakness	100 ~ 1,000	10 ~ 30	48 ~ 60
	Myotonia			
	Cataracts			
	Cardiac arrhythmias			
Congenital	Infantile hypotonia	> 1000	Birth to 10	45
	Respiratory failure			
	Intellectual disability			

The initial diagnosis of respiratory failure secondary to heart failure, which was made based on pre-existing severe hypertension, was incorrect. Instead, some differential diagnoses such as asthma attack and weakness of respiratory muscles could have been considered. The acute onset of respiratory failure was a clue for the possibility of an asthma attack in the present case because she had sometimes had asthma attacks in the past. However, the possibility of an asthma attack was ruled out in this case because the respiratory failure improved rapidly after intubation. In contrast, dyspnea progressed rapidly after extubation, which suggested weakness of the respiratory muscles and made the diagnosis difficult. It was thought that several conditions might have affected her respiratory failure.

Detailed interview about her past and family history on arrival at our hospital might have facilitated earlier, correct diagnosis and treatment. In addition, her physical findings and events in the ICU were distinctive. Her facial appearance and calvities were characteristic of myotonic dystrophy type 1, and circulatory events in the ICU were also keys for diagnosis. Some reports have suggested that fatal arrhythmias, such as ventricular tachycardia and Vf, and takotsubo syndrome are often clues for the diagnosis of myotonic dystrophy type 1 [4–6]. Since a genetic test is the only way to diagnose myotonic dystrophy type 1, we should not hesitate to perform genetic testing when we encounter a case that has difficulty recovering from respiratory failure.

## Conclusion

This case, which was newly diagnosed with myotonic dystrophy type 1 by genetic testing, is rare because the patient was middle-aged. However, some respiratory and circulatory events are key clues for making the diagnosis. When a neuromuscular disease such as myotonic dystrophy is suspected from physical findings and events, genetic testing should be performed.

## Data Availability

Not applicable due to patient privacy concerns.

## Abbreviations

CTG: Cytosine-thymine-guanine
DMPK: Dystrophia myotonica protein kinase
CNS: Central nervous system
ICU: Intensive care unit
Vf: Ventricular fibrillation
